# Identification of an Optimal Gyroid Microarchitecture of 3D-Printed Hydroxyapatite Bone Substitutes for Vertical Bone Augmentation and Osteoconduction

**DOI:** 10.3390/biom16070974

**Published:** 2026-07-02

**Authors:** Ekatarina Maevskaia, Julien Guerrero, Chafik Ghayor, Indranil Bhattacharya, Porawit Kamnoedboon, Franz E. Weber

**Affiliations:** 1 Center of Dental Medicine, Oral Biotechnology & Bioengineering, University of Zurich, 8032 Zurich, Switzerland; ekatarina.maevskaia@empa.ch (E.M.); julien.guerrero@zzm.uzh.ch (J.G.); chafik.ghayor@usz.ch (C.G.); indranil.bhattacharya@usz.ch (I.B.); porawit.k@chula.ac.th (P.K.); 2 Center for Applied Biotechnology and Molecular Medicine (CABMM), University of Zurich, 8057 Zurich, Switzerland; 3 Faculty of Dentistry, Chulalongkorn University, Bangkok 10330, Thailand

**Keywords:** gyroid, TPMS, microarchitecture, vertical bone augmentation, osteoconduction, bone substitute, additive manufacturing, 3D printing, ceramics

## Abstract

Triply periodic minimal surface (TPMS) microarchitectures combine low weight with high mechanical strength, and, in particular, G-gyroid-based microarchitectures represent a promising option for bone substitutes. Most studies on G-gyroid-based bone substitutes have reported only in silico or in vitro results, whereas in vivo data remain scarce and are generally limited to single G-gyroid microarchitectures. To identify the optimal G-gyroid microarchitecture for bone substitute applications, we compared three different G-gyroid designs with varying surface-to-surface distances to determine the most suitable architecture for osteoconduction and vertical bone augmentation. From a mechanical perspective, constructs with a wall-to-wall distance of 0.50 mm (gyroid05) exhibited higher compressive strength than those with distances of 0.80 mm (gyroid08) and 1.10 mm (gyroid11). In vivo assessment in a rabbit calvarial defect model demonstrated that defect bridging was improved by 44% with gyroid05 and by 40% with gyroid11 compared with gyroid08. In contrast, evaluation in a rabbit calvarial vertical bone augmentation model showed that bone height gain increased by 39% and 32% with gyroid08 and gyroid11, respectively, relative to gyroid05. Overall, the gyroid11 design demonstrated superior in vivo performance in defect bridging and bone augmentation, indicating that it may represent the most promising universal G-gyroid microarchitecture for bone substitutes.

## 1. Introduction

Triply periodic minimal surface (TPMS)-based constructs are characterized by periodicity in three dimensions, without self-intersections and by zero mean curvature, resulting in minimal weight combined with high mechanical strength [1,2]. TPMS-based architectures have been implemented in satellites, aircraft, and electric vehicles [3]. Their combination of low weight and favorable mechanical properties makes them particularly attractive for the design of bone substitutes to treat bone injuries [4]. Moreover, the tortuous structure of TPMS resembles trabecular bone [5]. Continuous surfaces with smooth transitions enhance cell adhesion and proliferation [6,7,8], thereby promoting bone tissue ingrowth [9]. In particular, the borderless interconnectivity facilitates rapid bone regeneration, vascularization, and material resorption [7,8].

Autologous bone remains the gold standard bone substitute in clinical practice for the treatment of bone injuries. Trabeculae form the basis of its microarchitecture and are continuously remodeled according to the mechanical load during normal bone turnover. This principle was first described by Wolff in 1892 and is known as Wolff’s law [10]. Attempts to translate this design principle into synthetic bone substitutes have often relied on the generation of pores using porogens or similar techniques [11]. However, the introduction of randomly distributed pores compromises the load-adapted architecture of bone and, by extension, its fundamental principle of combining low weight with high strength. This limitation was largely overcome with the advent of additive manufacturing [11,12], which enables the fabrication of ordered pore-based and other microarchitectures optimized for defect healing and osteoconductivity [13].

Histological studies from the early clinical use of autologous bone grafts more than 150 years ago have demonstrated that bone grows from the defect margins through the graft to bridge the defect. This phenomenon was termed osteoconduction by J. B. Murphy prior to World War I and was first described in the literature by Turner in 1914 [14]. Following the discovery of osteoinduction by Marshall Urist in 1965 [15], both processes required clear definition and distinction. According to Urist [16], osteoconduction is a three-dimensional (3D) process involving the ingrowth of capillaries, perivascular tissue, and osteoprogenitor cells from a bony bed into the porous structure of an implant, which serves as a guiding cue for bone regeneration. Osteoinduction, in contrast, refers to the recruitment and differentiation of uncommitted mesenchymal stem cells into osteoprogenitor cells, leading to bone formation even at heterotopic sites. A key difference between these processes is that osteoconduction requires a bony bed and occurs only at orthotopic sites, whereas osteoinduction can occur independently of pre-existing bone at any site. In osteoconduction, the scaffold primarily serves as a structural guide, whereas in osteoinduction, it additionally acts as a delivery system for osteoinductive factors such as bone morphogenetic proteins.

In the context of bone healing, rapid bone ingrowth and defect bridging (i.e., high osteoconductivity) are desirable scaffold properties. In dentistry, vertical bone augmentation is often required to provide sufficient bone volume for stable dental implant placement [17,18]. However, both osteoconduction and bone augmentation depend strongly on the microarchitecture of the scaffolds [19]. For pore-based microarchitectures, we previously identified an optimal pore size of 1.2 mm for osteoconduction and 1.7 mm for bone augmentation. When investigating TPMS-based designs, gyroid structures appeared particularly favorable for both osteoconduction and bone augmentation [20]. Gyroid-based microarchitectures produced by additive manufacturing have shown promising results in bone augmentation and osseointegration [21], as well as in the treatment of bone defects through enhanced osteoconduction [22].

Most studies investigating G-gyroid microarchitectures for bone substitute applications have reported only in silico or in vitro data, while relatively few have included in vivo results [23,24]. Furthermore, to our knowledge, no in vivo study has systematically compared different G-gyroid microarchitectures. To identify the optimal gyroid-based microarchitecture for osteoconduction and bone augmentation, we systematically varied the distance between the adjacent gyroid surfaces and fabricated three hydroxyapatite-based scaffolds with distinct G-gyroid microarchitectures using 3D printing. The scaffolds were evaluated over a period of 4 weeks in two well-established rabbit calvarial models. Bone ingrowth into a critical-size defect was assessed as a measure of osteoconduction, while vertical bone formation in an onlay configuration was used to evaluate bone augmentation.

## 2. Materials and Methods

### 2.1. Materials

#### 2.1.1. Manufacturing of Scaffolds

A lithography-based 3D printer for ceramics (CeraFab 7500, Lithoz, Vienna, Austria), fed with photosensitive hydroxyapatite-based slurry LithaBone^TM^ HA 400 (Lithoz, Vienna, Austria), was used to produce green bodies of HA-based scaffolds. The 3D-printed scaffolds were cleaned with pressured air and LithaSol 30^TM^ (Lithoz, Vienna, Austria). After cleaning, the scaffolds were debindered and sintered with a maximal temperature of 1300 °C. Stl files, containing coding for the scaffolds, were designed with the nTopology v.3 software (New York, NY, USA).

#### 2.1.2. Implant Designs

**Figure 1:**
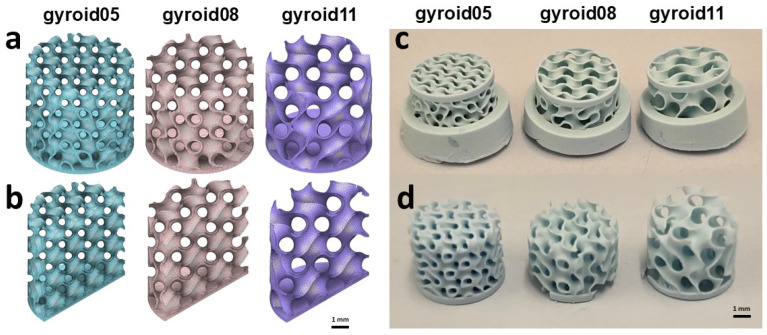
Macroarchitectures of the scaffolds. Overall macroarchitectures of full (**a**) and halved (**b**) scaffolds are illustrated. Scaffolds used for the cranial defect model are displayed in panel (**c**) and for bone augmentation in panel (**d**). Scale bars of 1 mm are provided.

The three gyroid based microarchitectures used for in vivo testing had a wall thickness of 0.2 mm and the surfaces were 0.5 mm, 0.8 mm, or 1.1 mm apart (Figure 1).

The overall design of scaffolds placed in defects to study osteoconduction had a diameter of 6.0 mm and a height of 5.0 mm. To restrict their advancement beyond the thickness of the calvarial bone, a solid outer ring with a 6.0 mm inner, 7.5 mm outer diameter, and a height of 2.5 mm was added to the upper part (Figure 1c). This feature restricted the advancement of the scaffold into the calvarial bone to 2.5 mm and stabilized the implant in the defect. Another ring, with an outer diameter of 6.0 mm, an inner diameter of 5.7 mm, and 0.5 mm in height, was added to the lower part (Figure 1c). The implants used to study vertical bone augmentation were identical to the ones for osteoconduction but lacked the solid outer ring (Figure 1d). The structural values for the different microarchitectures of the scaffolds are provided in Table 1.

### 2.2. Methods

#### 2.2.1. Surgical Procedure and Sample Preparation

Seventeen mature female New Zealand white rabbits (12 months old), weighing between 3.5 and 4.5 kg, were used for the two procedures. Each animal received all three treatment modalities plus one experimental scaffold. Both procedures were approved by the Animal Ethics Committee of the local authority (Veterinäramt, Canton Zurich, 090/2021) and in line with the ethics criteria contained in the bylaws of the Institutional Animal Care and Use Committee. Both procedures have been described in length in [19].

**Figure 2:**
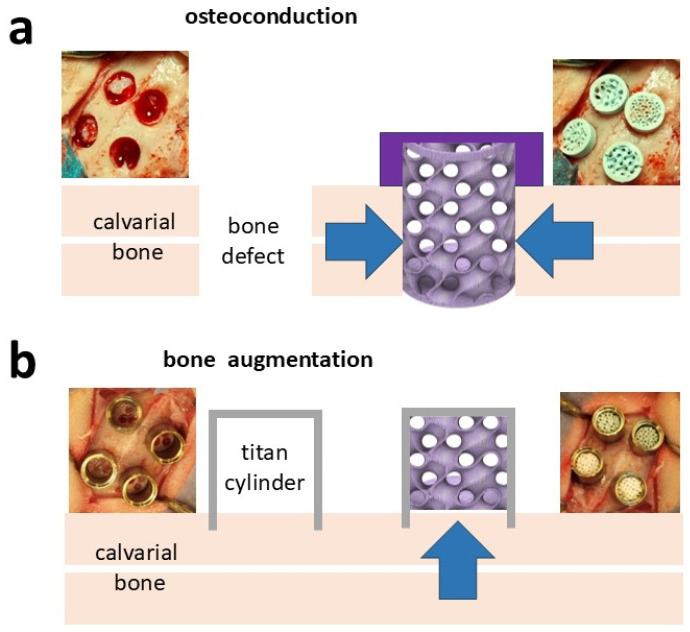
Schematic diagrams of the models for osteoconduction and bone augmentation. Panel (**a**), the calvarial defect model; panel (**b**), the bone augmentation model. In both panels, the left side is before implant placement and the right side after implant placement. The gray lines represent titanium cylinders. The blue arrows in (**a**,**b**) represent the bone ingrowth to occur in the osteoconduction/defect model from the right and left sides and in the bone augmentation model from one side only. The pictures on the left in (**a**,**b**) were taken just before implant placement and show the four 6 mm defects and the already installed titanium cylinders. The pictures on the right in (**a**,**b**) show the situation just after implant placement into the defects and the titanium cylinders.

Defect model in brief: An incision from the nasal bone to the mid-sagittal crest was made, the soft tissue carefully deflected, and the periosteum removed to expose the cranial bone. Four full-thickness craniotomy defects of 6 mm diameter were created, starting with a trephine bur followed by rose burs of 5 mm diameter, with 1 mm diameter to preserve the dura (Figure 2a, left side). Implants were applied by gentle press-fit placement (Figure 2a, right side) and the skin sutured to cover the calvarial bone and scaffolds.

Vertical bone augmentation model in brief: Four circular recipient sites (6.0 mm in diameter, 1.0 mm depth) were prepared in the calvarial bone using a trephine bur. To promote bone outgrowth from the underlying calvarium, the external cortical plate within each site was perforated three times using a 1.0 mm round bur. Titanium cylinders (7.0 mm in height and 7.0 mm in outer diameter) were then screwed into the prepared sites (Figure 2b, left side). The cylinders were filled with the corresponding scaffolds (Figure 2b, right side) and sealed with a titanium lid. Finally, the soft tissues were repositioned and sutured to achieve complete coverage of the calvarial bone and implanted cylinders.

#### 2.2.2. Histomorphometry

The central ground section of each implant, obtained from methyl methacrylate-embedded specimens, was analyzed using image analysis software (Image-Pro Plus^®^ 7.0; Media Cybernetics, Silver Spring, MD, USA) as described in [25]. For each sample, the section corresponding to the middle portion of the implant was selected for evaluation. In the defect model, the two halves of the 6 mm defect defined the area of interest (AOI). In the bone augmentation model, the AOI comprised the entire area within the titanium cylinder. The extent of bone ingrowth was measured from each defect margin toward the center of the scaffold, and in the augmentation model, from the basal interface between the cylinder and calvarial bone toward the most coronal point of newly formed bone. The percentage of bony regeneration was calculated as the area occupied by newly formed bone and bone-integrated scaffold within the AOI, divided by the total AOI area, and expressed as a percentage (area of bony regeneration, %).

#### 2.2.3. Osteoconduction and Vertical Bone Augmentation

Osteoconduction as measure of bony bridging of a defect was determined as previously reported [25]. Since vertical bone augmentation occurs from one side only, we measured one-sided osteoconduction as the stretch of bone ingrowth from each side of the defect margins in mm. For 100% bridging of the defect, the length of bone ingrowth from both sides was 3 mm (half the defect size). The area of interest in the defect model is set by the defect margins and the thickness of the calvarial bone. Vertical bone augmentation was defined as the maximum distance between the lowest end of the cylinder and the uppermost position where mineralized bone tissue was found [19].

#### 2.2.4. Mechanical Testing

For mechanical testing, the cubic models were designed with a side size of 7.80 mm. The universal testing machine ROELL Z2.5 MA 18-1-3/7 (ZwickRoell GmbH & Co KG, Ulm, Germany) was used to determine the mechanical properties of six scaffolds per group. The machine was set to apply compressive load at a speed of 1 mm/min in the direction of building layers. The software provided by the manufacturer of the machine (TestXpert V11.02 software (ZwickRoell GmbH & Co KGZwick, Ulm, Germany)) was used to determine the breaking point.

#### 2.2.5. Statistics

The defects, half of defects from the left side or right side, and the cylinders were the primary analysis units in this study. Data from 8 to 9 different rabbits were compiled and comprised groups of 8–9 samples. The different gyroid-based groups were compared using the Kruskal–Wallis test for defects and the Jonckheere–Terpstra test for bone augmentation and mechanical testing, followed by pairwise comparison using the Mann–Whitney test for independent data. Statistical analyses were performed using IBM SPSS Statistics version 19.0 (IBM Corp., Armonk, NY, USA). Statistical significance was set at a limit of *p* < 0.05. *p*-values are displayed in the graphs, and the data are presented in the text as mean ± standard deviation or as median ± lower/upper quartile.

## 3. Results

### 3.1. HA-Based Scaffolds for Bone Augmentation and Osteoconduction

All animals treated with scaffolds, either for bone augmentation or osteoconduction, remained in good health. Based on the behavior of the animals and the ground sections, indications of inflammation were absent. Irrespective of the gyroid design, bone formation occurred in the cylinders, and the defects close to the bone substitutes or on the surface of the scaffolds indicated a high biocompatibility with the HA-based material.

### 3.2. Mechanical Characteristics

**Figure 3:**
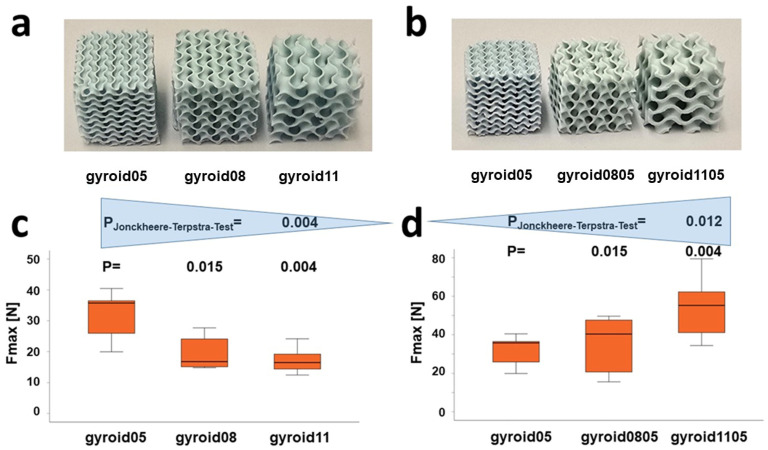
Mechanical testing. The samples used for the two test sets are shown in (**a**,**b**) with the maximal compression force in (**c**,**d**). The compression force decreases significantly with the reduction in the number of walls (**c**). The adjustment of the scaffolds’ material by increasing the wall thickness induced a significant increase in compression force. *p*-values are provided. The graphs in the lower panel visualize the results of testing for the maximal force in a box plot, ranging from the 25th (lower quartile) to the 75th (upper quartile) percentile, with the median displayed as a solid black line and whiskers extending to the minimum and maximum values. The sample size N for all different scaffolds was six.

Samples used for mechanical testing are displayed in Figure 3, together with the scaffolds’ compression strengths. We observed that the compression strength of the gyroids decreased significantly with the increase in distance between the walls from 32.37 ± 7.75 N for gyroid05 to 19.22 ± 5.37 N for gyroid08, and to 17.20 ± 4.12 N for gyroid11 (Figure 3c). Since an increase in the distance between the surfaces goes hand-in-hand with a decrease in material per volume, we produced a second set of scaffolds identical in material weight per volume by increasing the wall thicknesses accordingly. These scaffolds are shown in Figure 3b. The compression strength was 32.37 ± 7.75 N for gyroid05 and increased significantly to 35.69 ± 14.23 N for gyroid0805 (i.e., weight adjusted modified gyroid08 scaffold) and to 54.60 ± 16.17 N for gyroid1105 (i.e., weight adjusted modified gyroid11 scaffold) (Figure 3d).

### 3.3. Optimal Gyroid-Based Microarchitecture for Osteoconduction

**Figure 4:**
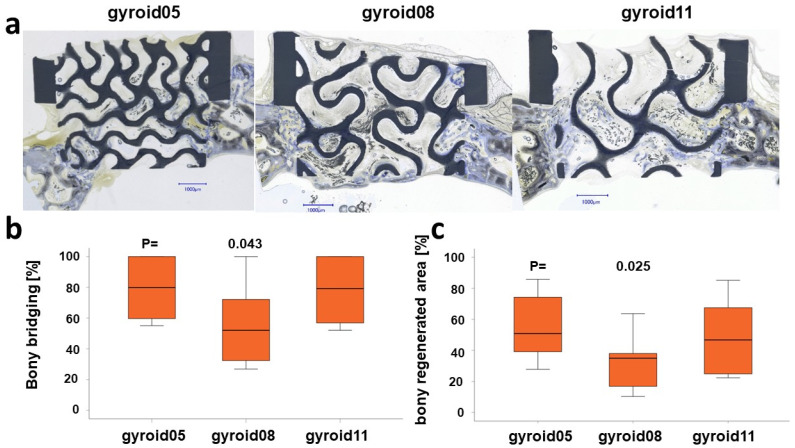
Osteoconduction and bone regeneration in gyroid-based microarchitectures. (**a**) The histological sections from the middle of noncritical-size defects were derived from samples harvested 4 weeks after operation. Scale bars in blue indicate 1000 µm. Original magnifications were 100-fold. Bone appears as grayish purple to purple, and HA as grayish to black. Percentage of defect bridging (**b**) and bony regeneration are (**c**) are shown. Values are displayed as box plots ranging from the 25th (lower quartile) to the 75th (upper quartile) percentile. The median is displayed as a solid black line and whiskers extending to the minimum and maximum values. *p*-values are provided in the graphs. Gyroid05 and gyroid11 performed equally well in defect bridging and bony regeneration. N for gyroid05 was 10, seven for gyroid08, and seven for gyroid11.

One aim of this study was to determine the osteoconductivity and bone regeneration capacity of three different gyroid-based scaffolds. Bony bridging and bony regeneration were determined with Toluidine Blue-stained ground sections from the middle of each scaffold (Figure 4a). The extent of bony bridging for gyroid05 was 79.85 ± 19.69%, 55.38 ± 28.71% for gyroid08, and 77.83 ± 22.45% for gyroid11 (Figure 4b). Gyroid05 performed significantly better than gyroid08. The *p*-value, however, was only 0.043. By using scaffolds based on gyroid05 or gyroid11 instead of gyroid08, bony bridging of the defect was, on average, increased by 44% and 40%, respectively.

For bony regenerated area, the percentage for gyroid05 was 55.05 ± 20.77%, 31.23 ± 18.37% for gyroid08, and 48.42 ± 25.61% for gyroid11 (Figure 4c). Gyroid05 performed significantly better than gyroid08 and equal to gyroid11. In the bony regenerated area, bone-to-implant contact was 38.52 ± 2.36% for gyroid05, 37.74 ± 2.34% for gyroid08, and 37.99 ± 2.36% for gyroid11 and was therefore identical for all three tested gyroid microarchitectures.

### 3.4. One-Sided Bone Ingrowth into Gyroid-Based Microarchitecture

**Figure 5:**
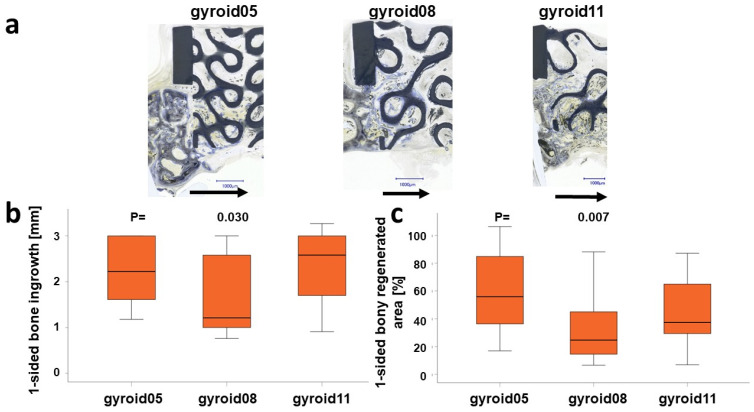
One-sided bone ingrowth into gyroid-based scaffolds. (**a**) central histologic sections of one-half of the 6 mm defects are shown. A scale bar for 1000 µm is provided. The stretch of maximal bone ingrowth is depicted by the black arrow. In the toluidine-stained sections, the scaffolds appear dark blue, and the bone appears grayish purple. One-sided bone ingrowth into the 6 mm defect (**b**) was similar for scaffolds gyroid05 and gyroid11. Gyroid05 appeared significantly better than gyroid08. Similar results were observed in terms of the percentage area of bony regeneration (**c**). Values are displayed as box plots ranging from the 25th (lower quartile) to the 75th (upper quartile) percentile, with the median displayed as a solid black line and the whiskers extending to the minimum and maximum values. *p*-values are displayed in the graph. N for gyroid05 was 20, 13 for gyroid08, and 13 for gyroid11.

Since bone augmentation occurs from one side only, we also determined one-sided bone ingrowth and regeneration in the defect model for osteoconduction. The one-sided advancement of bone tissue was 2.26 ± 0.70 mm for gyroid05, 1.69 ± 0.87 mm for gyroid08, and 2.34 ± 0.79 mm for gyroid11 (Figure 5b). In a direct comparison, the scaffolds gyroid05 and gyroid11 performed equally well, but gyroid05 was significantly better than gyroid08 (Figure 5b). Bony regenerated area (Figure 5c) for gyroid05 was 59.31 ± 27.77%, 33.25 ± 26.34% for gyroid08, and 44.35 ± 22.81% for gyroid11. For both measures, gyroid05 and gyroid11 performed equally well than gyroid08; however, gyroid05 was significantly better.

### 3.5. Optimal Gyroid-Based Microarchitecture for Vertical Bone Augmentation

**Figure 6:**
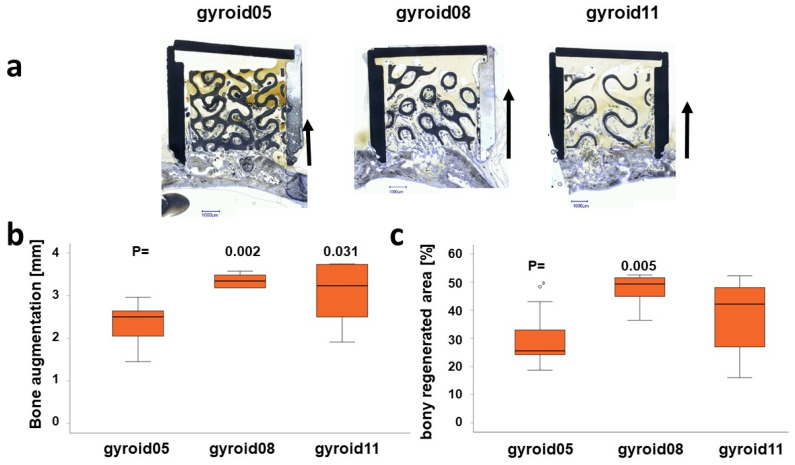
Vertical bone augmentation of gyroid-based scaffolds. (**a**) depicts the central histologic sections of cylinders harvested from the crania of rabbits after 4 weeks. The cylinders were filled with scaffolds gyroid05, gyroid08, or gyroid11. Advancment of bone ingrowth is depicted by black arrows. Titanium appears black, scaffolds appear dark blue, and bone appears grayish purple. Scale bares are provided. (**b**) bone augmentation and (**c**) bony regenerated area values are displayed as box plots ranging from the 25th (lower quartile) to the 75th (upper quartile) percentile, with the median displayed as a solid black line and the whiskers extending to the minimum and maximum values. *p*-values are displayed in the graph. N for gyroid05 was nine, six for gyroid08, and nine for gyroid11.

Over the 4-week implantation period, the bone front advanced vertically in all three microarchitectures. For gyroid05 scaffolds, a bone advancement of 2.35 ± 0.49 mm into the cylinders was observed. The advancement was 3.27 ± 0.30 mm for the gyroid08 and 3.10 ± 0.70 mm for the scaffold gyroid11 (Figure 6a,b). In a direct comparison of bone augmentation, scaffolds gyroid08 and gyroid11 performed significantly better than the scaffold gyroid05 (Figure 6b). For the area of bony regeneration (Figure 6c), gyroid08 performed significantly better than gyroid05. The area of bony regeneration was 30.39 ± 9.76% for gyroid05, 47.35 ± 6.38% for gyroid08, and 38.04 ± 13.76% for gyroid11 (Figure 6c). The use of gyroid08 or gyroid11 scaffolds instead of gyroid05 resulted in an average increase in vertical bone augmentation after 4 weeks of 39% and 32%, respectively.

## 4. Discussion

The superiority of gyroid microarchitectures over lattice-based and other triply periodic minimal surface (TPMS) microarchitectures for bone substitute applications has been demonstrated in several in vivo studies [26,27]. However, most investigations of gyroid-based bone substitutes have been limited to in silico or in vitro analyses. In vivo studies remain scarce and, where available, have generally focused on a single G-gyroid microarchitecture [28]. Consequently, knowledge regarding the optimal gyroid microarchitecture for bone substitute applications remains limited. To address this gap, we compared the performance of three gyroid-based microarchitectures, with wall spacings of 0.5 mm (gyroid05), 0.8 mm (gyroid08), and 1.1 mm (gyroid11). Their performance was evaluated in terms of compressive strength, vertical bone augmentation, and bone ingrowth into a bony defect as indicators of osteoconductivity. The latter two parameters are particularly important determinants of the clinical success of gyroid-based bone substitutes in orthopedic, cranio-maxillofacial, and dental applications.

Compression testing revealed that increasing the distance between the walls, which corresponds to a decrease in the number of walls per volume, resulted in a significant reduction in maximal compressive force. When the microarchitectures were normalized for material volume by increasing wall thickness, the scaffold with the thickest walls (gyroid1105) exhibited significantly higher compression strength than other scaffolds. By varying the scaffold microarchitecture through adjustments in wall number and wall thickness, compression strengths in the range of 28–90 MPa were achieved (Figure 3). The lower values are approximately twice as high as those reported for cancellous bone (2–12 MPa) [29] and the higher values approach the strength of cortical femoral bone in 70-year-old women (about 90 MPa) [30]. Therefore, variations in our gyroid design realized in 3D-printed HA-based scaffolds can potentially cover the full range of mechanical requirements for human bone replacement.

In TPMS-based microarchitectures, the 2D maximal sphere diameter has been identified as the most critical parameter for osteoconduction and should not exceed 1.53 mm [27]. All gyroid microarchitectures tested in this study fulfilled this criterion (Table 1). However, gyroid08 performed significantly worse than gyroid05 in terms of bony bridging and regenerated bone area (Figure 4). This suggests that another as-of-yet unidentified aspect of gyroid08’s design compared to gyroid05 may impair optimal osteoconductivity.

Bone ingrowth in a defect occurs from across the entire bony bed and is evident histologically from both sides (Figure 2b). In bone augmentation scenarios, however, bone growth occurs predominantly from one side only (Figure 2b). To make the ingrowth depth comparable, we also measured one-sided bone ingrowth in our defect model (Figure 5). Gyroid05 performed significantly better than gyroid08 in terms of one-sided ingrowth depth and regenerated bone area, while gyroid11 performed similarly to gyroid05. The maximal bone ingrowth depth in this comparison averaged 2.34 ± 0.79 mm for gyroid11. In the bone augmentation model, the maximal bone ingrowth depth was 3.27 ± 0.30 mm for gyroid08. Maximal bone advancement in an optimized honeycomb microarchitecture has been reported to be 3.1 ± 0.8 mm, which is in a similar range to [31]. A disadvantage of the honeycomb microarchitecture is that it allows bone ingrowth only in one direction and would therefore be unsuitable for our cranial defect model, where bone ingrowth occurs from multiple directions across the bony bed. The gyroid architecture has been shown to perform well in bone augmentation and onlay models [21], and also performed well in our and other’s defect models [32,33,34], suggesting broader applicability combined with low material intake and high strength.

In terms of vertical bone ingrowth and bone regeneration, gyroid08, and gyroid11 performed significantly better than gyroid05 (Figure 6). This is consistent with results obtained using pore-based microarchitectures, where larger pores (1.70 mm in diameter) also outperformed smaller pores [19]. However, achieving sufficient bone height for dental implant placement rarely requires vertical bone augmentation alone. In most cases, lateral bone ingrowth is also necessary. Therefore, the scaffold microarchitecture should promote both vertical and lateral bone ingrowth simultaneously. Our findings suggest that, for universal application of gyroid microarchitectures, the optimal distance between adjacent walls is approximately 1.10 mm, as gyroid11 performs well in both the bone augmentation (Figure 6) and the bone defect models (Figure 5). Its lower compression strength compared to gyroid05 or gyroid08 can be compensated by increasing the wall thickness (Figure 3d).

In the future, personalized bone substitutes based on the identified gyroid11 design could be manufactured using 3D printing and applied in orthopedics, cranio-maxillofacial surgery, and dentistry. Bone substitutes incorporating this optimized G-gyroid microarchitecture may also serve as delivery systems for biomolecules [28] and exosomes [35]. However, the selection of the gyroid11 design as the optimal architecture is currently based solely on results obtained in rabbits and therefore requires validation through clinical trials in humans. Because the material and methodology used in this study are already of medical-grade quality, progression to clinical evaluation may be feasible soon.

## 5. Conclusions

3D-printed hydroxyapatite (HA)-based bone substitutes with a gyroid microarchitecture are promising candidates for bone tissue engineering, as they combine low weight and reduced material usage with high mechanical strength. Optimization of gyroid architectures led to the identification of the gyroid11 design, characterized by a wall spacing of 1.10 mm, which demonstrated favorable performance in in vivo rabbit calvarial models with regard to bone regeneration, osteoconduction, and vertical bone augmentation. Given that variations in the gyroid11 architecture can be tailored to match mechanical properties, ranging from cancellous to cortical bone, functionally graded gyroid11 structures may represent versatile microarchitectures suitable for a wide range of clinical bone substitution applications in orthopedy, cranio-maxillofacial surgery, and dentistry.

## Figures and Tables

**Table 1:** Structural values for different scaffold microarchitectures.

Characteristics of Scaffolds	Area, [mm^2^]	Volume Material [mm^3^]	Area/ Volume Material [1/mm]	Porosity [%]	2D Maximal Sphere Diameter Fitting in Architecture [mm]
Gyroid05	639.54	38.72	16.52	70.67	0.55 ± 0.07
Gyroid08	515.40	30.96	16.65	76.55	0.75 ± 0.07
Gyroid11	395.98	23.43	16.90	82.25	1.20 ± 0.05

## Data Availability

The raw/processed data required to reproduce these findings cannot be shared at this time, as the data also form part of additional ongoing studies.

## Abbreviations

The following abbreviations are used in this manuscript:

TPMS	Triply Periodic Minimal Surface
HA	Hydroxyapatite
stl	Standard Triangle Language

## References

[1] Vijayavenkataraman S., Zhang L., Zhang S., Hsi Fuh J.Y., Lu W.F. (2018). Triply Periodic Minimal Surfaces Sheet Scaffolds for Tissue Engineering Applications: An Optimization Approach toward Biomimetic Scaffold Design. ACS Appl. Bio Mater..

[2] Yang L., Yan C., Han C., Chen P., Yang S., Shi Y. (2018). Mechanical response of a triply periodic minimal surface cellular structures manufactured by selective laser melting. Int. J. Mech. Sci..

[3] Feng J., Fu J., Yao X., He Y. (2022). Triply periodic minimal surface (TPMS) porous structures: From multi-scale design, precise additive manufacturing to multidisciplinary applications. Int. J. Extrem. Manuf..

[4] Dong Z., Zhao X. (2021). Application of TPMS structure in bone regeneration. Eng. Regen..

[5] Liu F., Ran Q., Zhao M., Zhang T., Zhang D.Z., Su Z. (2020). Additively Manufactured Continuous Cell-Size Gradient Porous Scaffolds: Pore Characteristics, Mechanical Properties and Biological Responses In Vitro. Materials.

[6] Yoo D.-J. (2014). Advanced porous scaffold design using multi-void triply periodic minimal surface models with high surface area to volume ratios. Int. J. Precis. Eng. Manuf..

[7] Elsheikh M., Kishida R., Hayashi K., Tsuchiya A., Shimabukuro M., Ishikawa K. (2022). Effects of pore interconnectivity on bone regeneration in carbonate apatite blocks. Regen. Biomater..

[8] Xia P., Luo Y. (2022). Vascularization in tissue engineering: The architecture cues of pores in scaffolds. J. Biomed. Mater. Res. Part B Appl. Biomater..

[9] Liu Y.-C., Lo G.-J., Shyu V.B.-H., Tsai C.-H., Chen C.-H., Chen C.-T. (2023). Surface Modification of Polylactic Acid Bioscaffold Fabricated via 3D Printing for Craniofacial Bone Tissue Engineering. Int. J. Mol. Sci..

[10] Wolff J. (1892). Das Gesetz der Transformation der Knochen.

[11] Hutmacher D.W. (2000). Scaffolds in tissue engineering bone and cartilage. Biomaterials.

[12] Jariwala S.H., Lewis G.S., Bushman Z.J., Adair J.H., Donahue H.J. (2015). 3D Printing of Personalized Artificial Bone Scaffolds. 3D Print. Addit. Manuf..

[13] Weber F.E. (2019). Reconsidering Osteoconduction in the Era of Additive Manufacturing. Tissue Eng. Part B Rev..

[14] Turner W.G. (1915). The Use of the Bone Graft in Surgery. Can. Med. Assoc. J..

[15] Urist M.R. (1965). Bone: Formation by autoinduction. Science.

[16] Urist M.R. (1976). Practical applications of basic research on bone graft physiology. The American Academy of Orthopaedic Surgeons: Instructional Course Lectures.

[17] Buser D., Dula K., Belser U.C., Hirt H.P., Berthold H. (1995). Localized ridge augmentation using guided bone regeneration. II. Surgical procedure in the mandible. Int. J. Periodontics Restor. Dent..

[18] Hämmerle C.H.F., Jung R.E., Yaman D., Lang N.P. (2008). Ridge augmentation by applying bioresorbable membranes and deproteinized bovine bone mineral: A report of twelve consecutive cases. Clin. Oral Implant. Res..

[19] Ghayor C., Bhattacharya I., Weber F.E. (2021). The optimal microarchitecture of 3D-printed β-TCP bone substitutes for vertical bone augmentation differs from that for osteoconduction. Mater. Des..

[20] Maevskaia E., Ghayor C., Bhattacharya I., Guerrero J., Weber F.E. (2024). TPMS Microarchitectures for Vertical Bone Augmentation and Osteoconduction: An In Vivo Study. Materials.

[21] Systermans S., Ronsmans C., Nolens G., Meyns J., Gilon Y., Cobraiville E. (2025). Dental Implant Osseointegration Following Mandibular Augmentation Using 3D Printed Hydroxyapatite Blocks. An Experimental In Vivo Study. Clin. Oral Implant. Res..

[22] Wang L., Wang Y., Liu R., Liang Y., Liu Y., Xu M., Yu J., Su Y., Han Z., Wang X. (2026). An Experimental Study on 3D-Printed Gyroid-Shaped TC4 Porous Scaffolds Guiding Angiogenesis and Osteogenesis in Bone Defect Areas. ACS Biomater. Sci. Eng..

[23] Budi M.N.S., Kariem M.A., Dwinata B., Hidayat Y.M., Sutiono A.B., Fathurachman F., Ismail W.F.N.W., Dwitama Y.G., Nugraha P. (2026). 3D-Printed PLA/HA Composite Scaffolds: Balancing Mechanical Properties for Bone Tissue Engineering. Materials.

[24] Li J., Fan H., Hua L., Du J., He Y., Jin Y. (2025). Bone implants with triply periodic minimal surface architectures: Design, fabrication, and biological performance. Bio-Des. Manuf..

[25] de Wild M., Amacher F., Bradbury C.R., Molenberg A. (2016). Investigation of structural resorption behavior of biphasic bioceramics with help of gravimetry, μCT, SEM, and XRD. J. Biomed. Mater. Res. Part B Appl. Biomater..

[26] Hayashi K., Kishida R., Tsuchiya A., Ishikawa K. (2023). Superiority of Triply Periodic Minimal Surface Gyroid Structure to Strut-Based Grid Structure in Both Strength and Bone Regeneration. ACS Appl. Mater. Interfaces.

[27] Maevskaia E., Guerrero J., Ghayor C., Bhattacharya I., Weber F.E. (2023). Triply Periodic Minimal Surface-Based Scaffolds for Bone Tissue Engineering: A Mechanical, and Study. Tissue Eng. Part A.

[28] Yan J., Qi S., Zhao Y., Tian P., Kong N., Ma W., Yan P., Zhang J., Gao X., Guan H. (2025). 3D-Printed Triply Periodic Minimal Surface Ceramic Scaffold Loaded with Bone Morphogenetic Protein-2 and Zoledronic for Cranium Defect Repairment. J. Tissue Eng. Regen. Med..

[29] Ben-Arfa B.A.E., Neto S., Miranda Salvado I.M., Pullar R.C., Ferreira J.M.F. (2019). Robocasting of Cu^2+^ & La^3+^ doped sol-gel glass scaffolds with greatly enhanced mechanical properties: Compressive strength up to 14 MPa. Acta Biomater..

[30] Havaldar R., Pilli S.C., Putti B.B. (2014). Insights into the effects of tensile and compressive loadings on human femur bone. Adv. Biomed. Res..

[31] Hayashi K., Shimabukuro M., Kishida R., Tsuchiya A., Ishikawa K. (2022). Structurally optimized honeycomb scaffolds with outstanding ability for vertical bone augmentation. J. Adv. Res..

[32] Hou B., Li Y., Wong R.C.W. (2026). Next-generation craniomaxillofacial implants for reconstructive surgery: Balancing biomechanics, biocompatibility, and bioactivity. Int. J. Oral Sci..

[33] Verisqa F., Park J.-H., Mandakhbayar N., Cha J.-R., Nguyen L., Kim H.-W., Knowles J.C. (2024). In Vivo Osteogenic and Angiogenic Properties of a 3D-Printed Isosorbide-Based Gyroid Scaffold Manufactured via Digital Light Processing. Biomedicines.

[34] Zhu H., Li M., Huang X., Qi D., Nogueira L.P., Yuan X., Liu W., Lei Z., Jiang J., Dai H. (2021). 3D printed tricalcium phosphate-bioglass scaffold with gyroid structure enhance bone ingrowth in challenging bone defect treatment. Appl. Mater. Today.

[35] Maevskaia E., Guerrero J., Ghayor C., Bhattacharya I., Weber F.E. (2024). Functionalization of Ceramic Scaffolds with Exosomes from Bone Marrow Mesenchymal Stromal Cells for Bone Tissue Engineering. Int. J. Mol. Sci..

